# Click gold quantum dots biosynthesis with conjugation of quercetin for adenocarcinoma exertion†

**DOI:** 10.1039/d2ra02529a

**Published:** 2022-06-23

**Authors:** Amol V. Pansare, Priyanka V. Pansare, Amol A. Shedge, Shubham V. Pansare, Vishwanath R. Patil, Giovanni P. Terrasi, Kamini J. Donde

**Affiliations:** Composite Group, Mechanical Systems Engineering, Swiss Federal Laboratories for Materials Science and Technology-Empa 8600 Dübendorf Switzerland giovanni.terrasi@empa.ch amol.pansare@empa.ch; Department of Chemistry, University of Mumbai Santacruz (E) Mumbai 400098 India vishwanathrpatil03@gmail.com; Ramnarain Ruia Autonomous College, University of Mumbai Matunga (E) India kaminidonde@ruiacollege.edu

## Abstract

We developed a cost-effective and eco-friendly click biosynthesis of small molecule quercetin–gold quantum dots (QRT–AuQDs) involving quick conjugation using an ultrasonication method at ambient temperature by utilizing QRT and gold ions in the proportion of 0.1 : 1 (molar ratio). A comparatively very short amount of time (60 seconds) was required as compared to conventional procedures. The present biomimetics research relates to the isolation of bioactive QRT by the circularly spread silica gel layer technique (CSSGLT) and characterization (UV-Vis, FTIR, NMR and DSC analysis). Characterization of the synthesized QRT–AuQDs conjugated complex was carried out by UV-Vis, HR-TEM, DLS, zeta potential and X-ray diffraction. The main objective of the present work was to study the comparative anticancer activity of QRT and QRT–AuQDs on human lung cancer HOP-62 and leukemia K-562 cell lines. The results suggested that QRT–AuQDs showed potential for applications in anticancer treatment and were found to be a more cytotoxic agent in comparison to QRT, causing > 50% inhibition of cancer cells at the concentration < 10^−7^ M. Hence, small molecule conjugated QRT–AuQDs can be used as a promising material for biomedical, bioengineering and anti-infectives applications.

## Abstract 2

A cost-effective and eco-friendly click biosynthesis of small molecule quercetin–gold quantum dots (QRT–AuQDs) was developed involving quick conjugation, using an ultrasonication method at ambient temperature by utilizing QRT and gold ions in the molar ratio 0.1 : 1.

## Graphical abstract

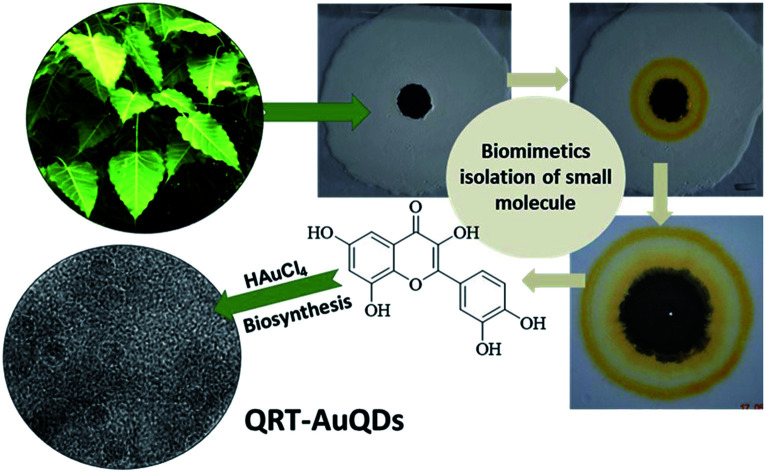

## 1. Introduction

The last decade has seen the proliferation of an enormous number of scientific studies focused on the activity of non-nutritional compounds present in the diet, which are able to prevent the occurrence of degenerative diseases, such as cancer and cardiovascular pathologies. This heterogeneous class of molecules, generally known as phytochemicals, includes vitamins and food polyphenols, such as flavonoids, phytoalexins, phenolic acids, indoles and sulfur-rich compounds.^1–3^

They are widely present in fruit, vegetables and beverages and are found in many dietary supplements and herbal remedies. However, what largely attracts scientists' interest is the number of compounds available for testing, with more than 10 000 phytochemicals potentially present in nature. The wide range of biological activities remains uncharacterized for most compounds.^4,5^

Phytochemicals, in fact, trigger cellular pathways that lead to the prevention or amelioration of pathological conditions associated with cancers, cardiovascular and neurodegenerative diseases.^6^

Although this positive association is debated and is the subject of criticism,^7^ biochemical and genetic studies on cellular and animal models on the mechanism of action of phytochemicals provide a functional explanation of how and why a diet rich in fruit and vegetables can protect against degenerative diseases.^8^

As well as the positive health effects of flavonoids, there have been some cautionary reports in the literature that raise concerns about potential side effects of excessive flavonoid intake.^9^ Flavonoids possess strong anti-oxidant activity and have been found to be therapeutically beneficial in diseases such as cancer and heart diseases.^10^ Quercetin is the most abundant dietary flavonoid in fruit and vegetables.^11^ According to the literature, the daily intake of quercetin varies widely. In the western world, the average daily intake of quercetin has been estimated to be between 20 mg and 40 mg, although it can increase up to 500 mg day^−1^ in individuals who ingest large quantities of apples, onions and tomatoes. Furthermore, quercetin is available as a dietary supplement, and ingestion of 1 g day^−1^ or even more has been reported.^12–15^ Quercetin is a dietary antioxidant that prevents oxidation of low-density lipoproteins *in vitro*. Intake of quercetin was inversely associated with coronary heart disease mortality in elderly dutch men.^16^ Several studies have shown that quercetin and other flavonoids have many therapeutically relevant properties, such as induction of apoptosis in tumor cells, and antiviral, antioxidant, anti-inflammatory and antiproliferative activities.^17–21^ It is also known to regulate blood glucose and insulins level in diabetic condition.^22–24^

Biological substances like black tea leaf extract and Indian propolis have been used for synthesis of gold nanoparticles (GNPs). There have been several reports on complexation of GNPs with different substances like proteins, DNA and carbon nanotubes.^25–29^

It is, therefore, expected that the gold nanoparticles–QRT complex may serve as a better therapeutic agent in nanomedicine. Researchers have reported the synthesis of the AuNPs–quercetin complex by using NaBH_4_ as the reductant,^24,30,31^ however, characterization of this AuQDs–quercetin complex has not been reported.

Due to its potentially beneficial impact on human health, the small molecule polyphenol QRT has come into the focus of medicinal interest. Hence, there is a need to isolate QRT from *F. Arnottiana* leaves, which represents the most abundant dietary flavonoid found in a broad range of fruit, vegetables and beverages, whose antioxidant and anti-inflammatory properties have been associated with the prevention and cancer therapy.

The main drawbacks of common synthesis routes for drug–metal complex are the employment of toxic chemicals and high temperatures,^32–34^ pressure and energy,^35–38^ so that new green and environmentally sustainable friendly strategies are needed.

Hence, as a continued effort, in the present work, we have isolated QRT from *Ficus (F) Arnottiana* plant leaves by CSSGLT and further synthesized the QRT–AuQDs complex by the ultra-sonication method. We comparatively evaluated the inhibitory potency of QRT and QRT–AuQDs against human lung cancer HOP-62 and leukemia K-562 cell lines by the SRB assay method.^39,40^

## 2. Experimental section

Alumina active basic was purchased from Merck Ltd India. Chloroauric acid, RPMI1640 medium, fetal bovine serum (FBS), l-glutamine, SRB, trichloroacetic acid (TCA) and trizma base were procured from Sigma-Aldrich. HOP-62 (lung cancer cell line) and K-562 (leukemia cancer cell line) were purchased from National Centre for Cell Science (NCCS), Pune, India. All anti-cancer activity experiments were performed at Tata Memorial Centre Advanced Centre for Treatment, Research & Education in Cancer (ACTREC), Mumbai, India. All chemicals were of AR grade. Glassware was properly washed with deionized water and dried in the oven.

### 2.1 Isolation of small molecule QRT by CSSGLT

A chromatographic separation was carried out using circularly spread silica gel on a glass plate (Fig. S1 and S2†). An ethanol extract of *F. Arnottiana* leaves was placed at the centre of the CSSGLT. On application of the 1st mobile phase (petroleum ether), separation of a dark yellow-colored band occurred from the centre of the silica gel circle. This band was removed by scratching, and the scratched area was filled up with fresh silica gel again. Then, the 2nd mobile phase (petroleum ether and chloroform in a 1 : 1 ratio) was used and a dark yellow-colored band was separated from the centre of silica gel circle. The 3rd mobile phase (chloroform) was used to separate a yellow-colored broad circle from the middle of the ethanol extract. This dark yellow-colored part was scratched and picked up from the spread silica gel in circular form, and fresh silica gel was filled up again in the scratched area. The 4th mobile phase (chloroform and acetone in a 2 : 1 ratio) was used to separate the colorless broad circle from the middle of the ethanol extract. This colorless part was scratched and picked up from the spread silica gel and the scratched area was filled up by aluminum oxide active base. Finally, the 5th mobile phase (acetone and methanol in a 2 : 1 ratio) was used and a dark yellow-colored circular band was separated from the middle of the ethanol extract. This dark yellow-colored fraction was further subjected to a pencil column using acetone : methanol (1 : 1) as the 6th mobile phase, from which three major fractions were separated out. Fraction 1 was a white-colored powder. Fraction 2 was a yellow-colored sticky mass. Fraction 3 was a yellow-colored powder.

### 2.2 Synthesis of small molecule QRT–AuQDs

QRT (0.1 M) was dissolved in 25 mL of acetone : methanol (1 : 1). Then, HAuCl_4_ (0.1 M) dissolved in 25 mL of methanol was added slowly. The reaction mixture was ultrasonicated at room temperature for 1 min and the solvent was then evaporated. The sample was collected, washed with 1 : 1 chloroform/*n*-butanol and then finally with water to remove the unreacted molecules. The QRT–AuQDs was dried under vacuum.

### 2.3 Characterizations of small molecule QRT

The physical state of the CSSGLT isolated QRT was characterized using DSC thermogram analysis (Mettler Toledo DSC 822e; India). 2.93 mg of QRT was weighed separately in standard aluminum pans. A DSC instrument was used for measurement of the melting temperature of QRT with an empty reference aluminum pan. The sample was purged in the DSC with pure dry nitrogen gas which was set at a flow rate of 40 mL min^−1^. The change in temperature was set at 5 °C min^−1^, and the heat flow was recorded from 25 °C to 400 °C. Heat flow was measured by comparing the difference in temperature across the sample and the reference. UV-visible spectroscopic analysis was performed using LAB UV3000^plus^ at room temperature with a quartz cuvette of 1 cm path-length as the sample holder (200–800 nm). IR spectra were recorded in the transmittance range of 4000 to 400 cm^−1^ on PerkinElmer FTIR spectrometer Frontier using ATR. The ^1^H and ^13^C NMR spectra of the QRT were recorded at 500 mHz (^1^H NMR) and 125 mHz (13C NMR) using the Bruker (Advance) NMR instrument in DMSO solvent.^41^^1^H NMR (500 MHZ, DMSO-*d*_6_): *δ* 6.18 (d, 1H, *J* = 2 Hz), 6.40 (d, 1H, *J* = 1.5 Hz), 6.87 (d, 1H, *J* = 9 Hz), 7.52 (dd, 1H, *J* = 2.5 and 8.5 Hz), and 7.67 (d, 1H, *J* = 2.5 Hz). ^13^C NMR (125 MHZ, DMSO-*d*_6_): *δ* 93.32, 98.15, 102.98, 115.03, 115.57, 119.94, 121.92, 135.70, 145.03, 146.77, 147.67, 156.11, 160.69, 163.85, and 175.81.

### 2.4 Characterization of small molecule QRT–AuQDs

The size range and zeta potential of the particles were investigated using dynamic light scattering (DLS) [Malvern, Instrument Ltd]. The small molecule QRT and QRT–AuQDs were characterized by imaging/spectroscopy techniques like UV-vis spectroscopy. The X-ray diffraction patterns of the QRT and QRT–AuQDs were recorded using a Shimadzu maxima 7000 X-ray diffractometer with CuKα radiation (*λ* = 0.154060 nm) with the scanning range between 10° to 100° to determine their crystalline nature.^42^ The surface morphology of QRT–AuQDs was probed by HR-TEM [FEI TECNAI TF-30 (FEG)].

### 2.5 *In vitro* cytotoxicity studies

#### 2.5.1 Cell culture

The human lung cancer cell line HOP-62 and human leukemia cancer cell line K-562 were propagated in RPMI1640 medium containing 10% fetal bovine serum and 2 mM l-glutamine at 37 °C humidified atmosphere of 5% CO_2_ in air.

### 2.6 *In vitro* anti-cancer activity study

A sulphorhodamine B (SRB) based *in vitro* cytotoxicity assay was performed to compare the anti-tumor effects of small molecule QRT, QRT–AuQDs and adriamycin (ADR positive control drug) against HOP-62 and K-562 cell lines according to the previously established method.^43^ HOP-62 and K-562 cells in the logarithmic phase were dispensed into 96 well microtiter plates in 90 μL at plating densities of 5 × 10^3^ cells per well. Different concentrations of small molecule QRT, QRT–AuQDs and ADR *viz.* 1 × 10^−7^ M, 1.0 × 10^−6^ M, 1.0 × 10^−5^ M and 1.0 × 10^−4^ M were prepared by serial dilution of the stock solution. The HOP-62 and K-562 cells were grown in plates for 24 hours and then compounds were added at different dilutions. Cultures were further incubated at standard conditions for 48 hours and the assay was terminated by the addition of cold TCA. Cells were fixed *in situ* by the gentle addition of 50 μL of cold 30% (w/v) TCA (final concentration, 10% TCA) and incubated for 60 min at 4 °C. The supernatant was discarded. The plates were washed five times with double distilled water and dried in air.

### 2.7 SRB staining

SRB solution (50 μL) at 0.4% (w/v) in 1% acetic acid was added to each of the wells, and plates were incubated for 20 minutes at room temperature. After staining, the unbound dye was recovered and the residual dye was removed by washing five times with 1% acetic acid. The plates were air dried. The bound stain was subsequently eluted with 10 mM trizma base, and the absorbance was read on an ELISA plate reader at a wavelength of 540 nm with 690 nm as a reference wavelength.^44^

## 3. Results and discussion

### 3.1 Characterization of small molecule QRT

The small molecule QRT (a yellow-colored powder-fraction 3) was isolated by CSSGLT from the *F. Arnottiana* leaves using the acetone : methanol (2 : 1) fraction of the ethanol extract. The DSC thermogram is shown in Fig. S3.†

The DSC curve of the isolated QRT exhibited two endothermic responses corresponding to its dehydration temperature (140 °C) and melting point (324 °C), followed by rapid decomposition.^45^ The UV-vis spectrum showed maxima at *λ*_max_ 254 nm (abs = 0.72) and 370 nm (abs = 0.84) suggesting a QRT chromophore (Fig. S4†).

The FTIR spectrum of QRT is shown in Fig. S5.† The major characteristic broader bands correspond to the OH phenolic stretch at 3410 cm^−1^ and the C=O absorption band at 1667 cm^−1^. The bands corresponding to its benzene ring can be observed at 1612, 1560 and 1523 cm^−1^, the C–H bending is at 1450 cm^−1^, and the region for the C–O stretch is at 1259 cm^−1^. These results are similar to previous reports.^46,47^

The ^1^H NMR for the QRT showed four doublets and one singlet. In the spectrum, three doublet signals appeared at *δ* 6.18, 6.40 and 7.67 ppm for the three aromatic protons due to meta coupling (Fig. S6†). Furthermore, the one proton resonating at *δ* 6.87 ppm showed ortho coupling and the remaining one proton exhibited a doublet of doublets at *δ* 7.52 ppm for the ortho and meta couplings.^48,49^ In the ^13^C-NMR spectrum of QRT (Fig. S7†), fifteen carbon signals were observed. Furthermore, the signals resonating at *δ* 175.81 ppm corresponded to the carbonyl carbon.

**Fig. 1:**
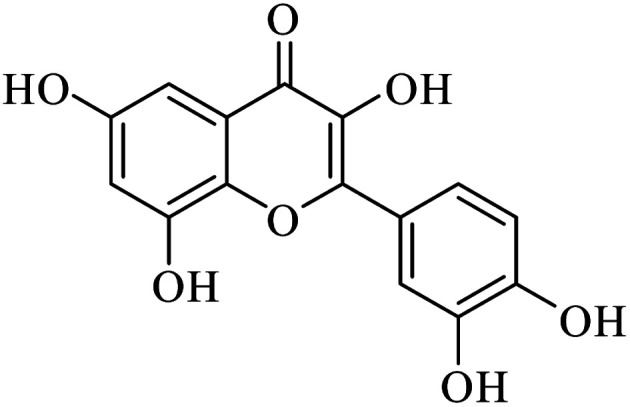
Structural formula for quercetin isolated from *F. Arnottiana*.

The signals resonating at *δ* 93.32 and 98.15 ppm were for the alkene carbon. However, other carbon signals were observed in the range of *δ* 102 to 163 for the eleven aromatic carbons. On the basis of the above spectral and chemical evidence, the isolated compound identified was QRT (Fig. 1).

### 3.2 Characterization of QRT–AuQDs

**Fig. 2:**
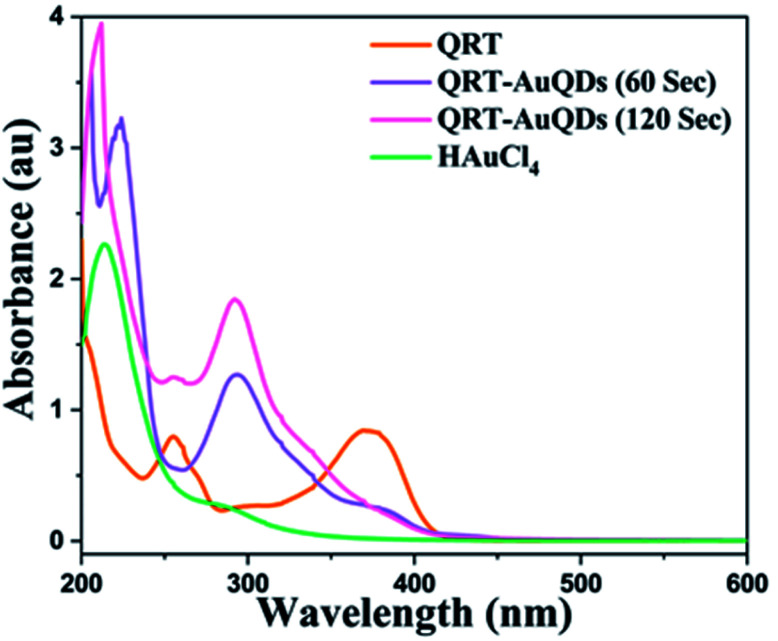
UV-vis absorption spectra of QRT, HAuCl_4_ and QRT–AuQDs.

The synthesized QRT–AuQDs complex was brownish pink in color and was stable at room temperature. The QRT–AuQDs complex was found to be soluble in DMSO and DMF but was sparingly soluble in methanol and ethanol and insoluble in water, *n*-hexane and DCM solvent. The free QRT in methanol solution showed two absorption peaks at 254 nm and 374 nm related to the conjugation. The UV-vis spectrum clearly suggested that formation of QRT–AuQDs in a 1 min time period. The absorbance of QRT–AuQDs increases at 294 nm, showing the characteristic peak, which is indicative of complex formation and is presented in Fig. 2.

**Fig. 3:**
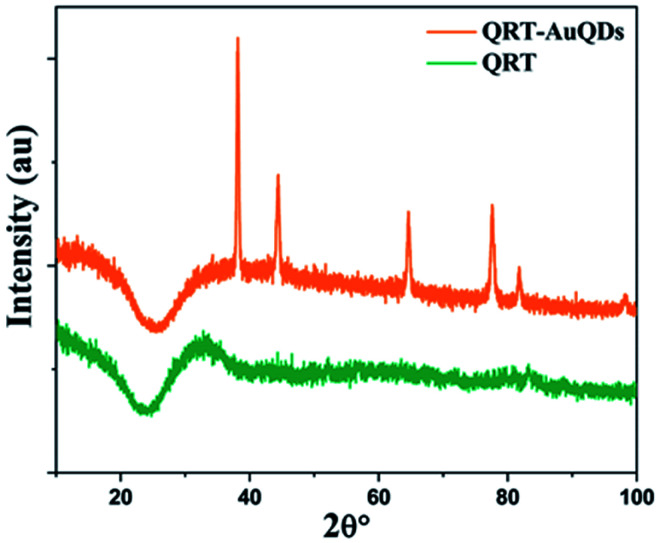
X-ray diffractograms of QRT and QRT–AuQDs.

**Fig. 4:**
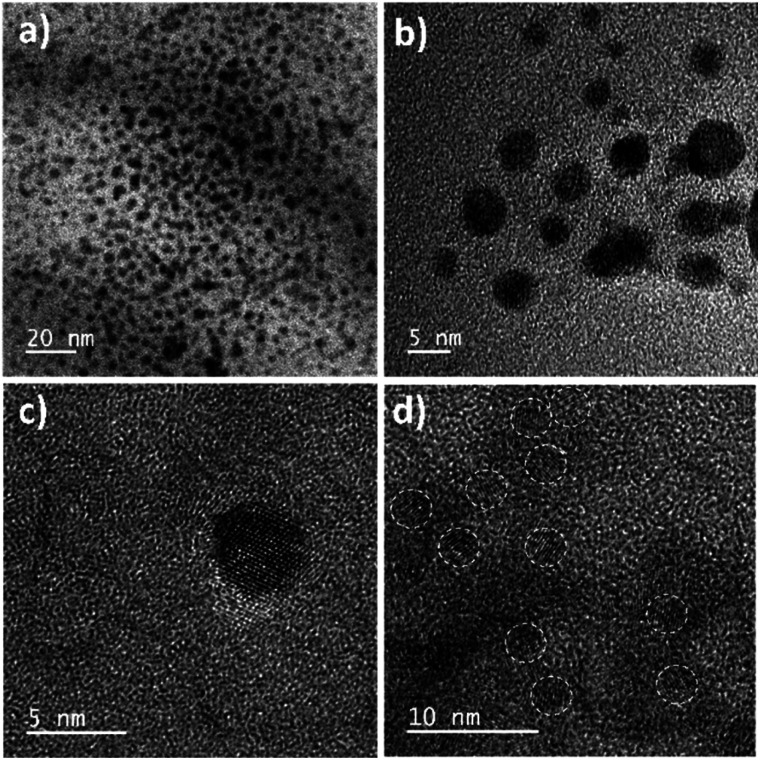
HR-TEM images of QRT–AuQDs.

The size distribution and average particle size of QRT–AuQDs were obtained using DLS. The results revealed in the histogram indicate that the average particle size (Fig. S8†) with maximum intensity was found to be at 4 nm. The zeta potential of about −47.0 mV was observed which indicated that QRT–AuQDs were stable in the colloidal state (Fig. S9†). In the FTIR spectrum of QRT–AuQDs (Fig. S10†), there was no measured change observed in the comparison with the FTIR spectrum of QRT (Fig. S5†), the broader band OH phenolic stretching at 3410 cm^−1^ intensity was lower in the comparison with QRT because of the consumption of hydroxyl groups during the formation of the QRT–AuQDs complex. X-ray powder diffractograms of QRT and QRT–AuQDs are shown in Fig. 3. The XRD pattern of QRT showed the characteristics of a slightly crystalline nature. In short, diffraction peaks were observed in the QRT–Au complex compared to free QRT. This finding was consistent with providing evidence that free QRT in the QRT–AuQDs was indeed converted to the crystalline state. The HR-TEM data revealed that Au is in its nano form and is physically bonded to QRT (Fig. 4).

### 3.3 Applications

#### 3.3.1 *In vitro* anti-cancer activity of the QRT–AuQDs

**Fig. 5:**
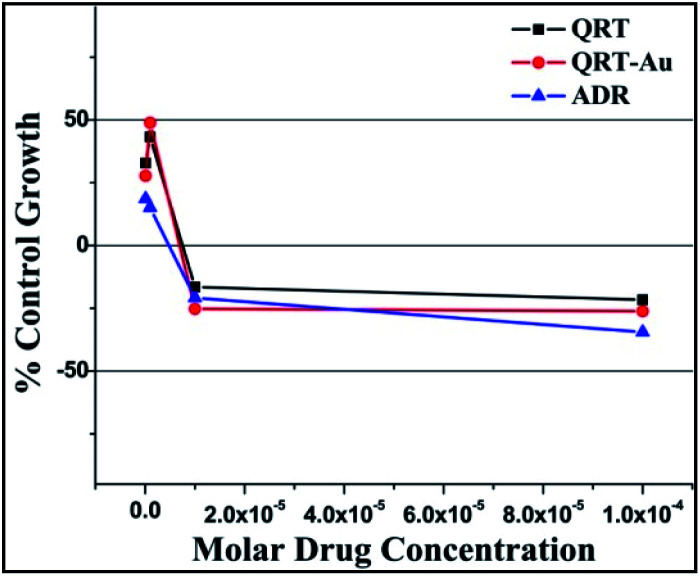
% Control growth curve of human lung cancer cell line HOP-62 with QRT, QRT–AuQDs and ADR positive control compound in molar concentrations.

**Fig. 6:**
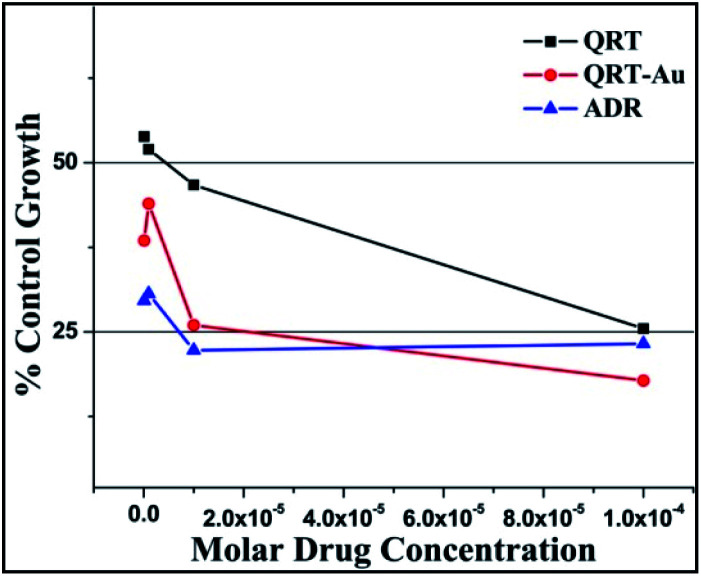
% Control growth curve of human leukemia cancer cell line K-562 with QRT, QRT–AuQDs and adriamycin (ADR) positive control compound in molar concentrations.

The *in vitro* anticancer activities of the small molecule QRT and QRT–AuQDs against adenocarcinoma human lung cancer cell line HOP-62 and leukemia cancer cell line K-562 were investigated in terms of GI_50_ (concentration of drug required to decrease the cell growth to 50%, compared with that of the untreated cell number), TGI (concentration of drug required to decrease the cell growth to 100%, compared with that of the untreated cell number during drug incubation) and LC_50_ (concentration of drug required to decrease the cell growth by 50% of the initial cell number prior to the drug incubation) values. All the assays were performed three times at various concentrations. Tables S1 and S2† contain the cytotoxicity data obtained by the SRB assay. The GI_50_ values were determined as the concentration of tested agents producing 50% decrease of cell survival. It was observed that the QRT–Au complex was found to be a more active cytotoxic agent in comparison to free QRT causing > 50% inhibition of HOP-62 and K-562 cell proliferation at concentrations < 10^−7^ M; QRT did not show >50% inhibition of K-562 cell proliferation at concentrations < 10^−7^ M. The results represented in Fig. 5, 6 and Table 1 and 2 demonstrated that green synthesized QRT–AuQDs have statistically significant anti-cancer activities against HOP-62 and K-562 and showed comparable IC_50_ values with ADR, a standard anti-cancer drug.

**Table 1:** HOP-62 human lung cancer cell line activity of QRT and QRT–AuQDs^a^

	Drug concentrations (μmolar) calculated from graph		
HOP-62	LC_50_	TGI	GI_50_*
QRT	>100	50.43	<0.1
QRT–AuQDs	>100	40.47	<0.1
ADR	<0.1	15.2	<0.1

a LC_50_ = concentration of drug causing 50% cell death, GI_50_ = concentration of drug causing 50% inhibition of cell growth, TG = concentration of drug causing total inhibition of cell growth, ADR = adriamycin, the positive control compound, a GI_50_ value of ≤10^−6^ molar (*i.e.* 1 μmolar) is considered to demonstrate activity.^50–52^

**Table 2:** K-562 human leukemia cell line activity of QRT and QRT–AuQDs

	Drug concentrations (μmolar) calculated from graph		
K-562	LC_50_	TGI	GI_50_*
QRT	>100	>100	7.22
QRT–AuQDs	>100	<0.1	<0.1
ADR	>100	>100	<0.1

## 4. Conclusions

This newly developed method describes the isolation of small molecule QRT by CSSGLT from *F. arnottiana* leaves for the first time and it requires lower quantities of solvent than the conventional route of column separation. The characterization data of the leaves extract confirmed the purity of the QRT fraction. We further successfully click biosynthesized QRT–AuQDs from isolated small molecule QRT and gold ions in a fraction of a minute. The QRT–AuQDs complex exhibited statistically significant anticarcinomic activities against HOP-62 and K562 in comparison to isolated small molecule QRT. Its observed GI_50_ value was comparable to ADR. The present method of isolation and characterization of bioactive QRT from the leaves of *F. Arnottiana* can be of great help for its standardization.

Hence, the method reported in the present biomimetics investigation is simple, easy and eco-friendly for the synthesis of the anticancerous small molecule QRT–AuQDs complex which could be recognized as a promising material for biomedical applications. Furthermore, we also need to elucidate the bioactivity, sensing behaviour of DNA and protein (BSA) and hemoglobin interactions of the small molecule QRT–AuQDs thoroughly.

## Conflicts of interest

The authors certify that there is no conflict of interest for our work.

## Supplementary Material

RA-012-D2RA02529A-s001
