# Supplementary material for: Click gold quantum dots biosynthesis with conjugation of quercetin for adenocarcinoma exertion
Source: RSC Adv. 2022 Jun 23;12(29):18425–30. doi: 10.1039/d2ra02529a (PMC9218964; doi:10.1039/d2ra02529a)
Supplement: RA-012-D2RA02529A-s001 [file RA-012-D2RA02529A-s001.pdf]

Electronic Supplementary Material (ESI) for RSC Advances.
This journal is © The Royal Society of Chemistry 2022

Supplementary Information

Click Gold Quantum Dots Biosynthesis with Conjugation of Quercetin for Adenocarcinoma Exertion

Amol V. Pansare[‡a,b], Priyanka V. Pansare[‡c], Amol A. Shedge[b], Shubham V. Pansare[b], Vishwanath R. Patil[b]*, Giovanni P. Terrasi [a]* and Kamini J. Donde[c]*

‡ Authors contributed equally

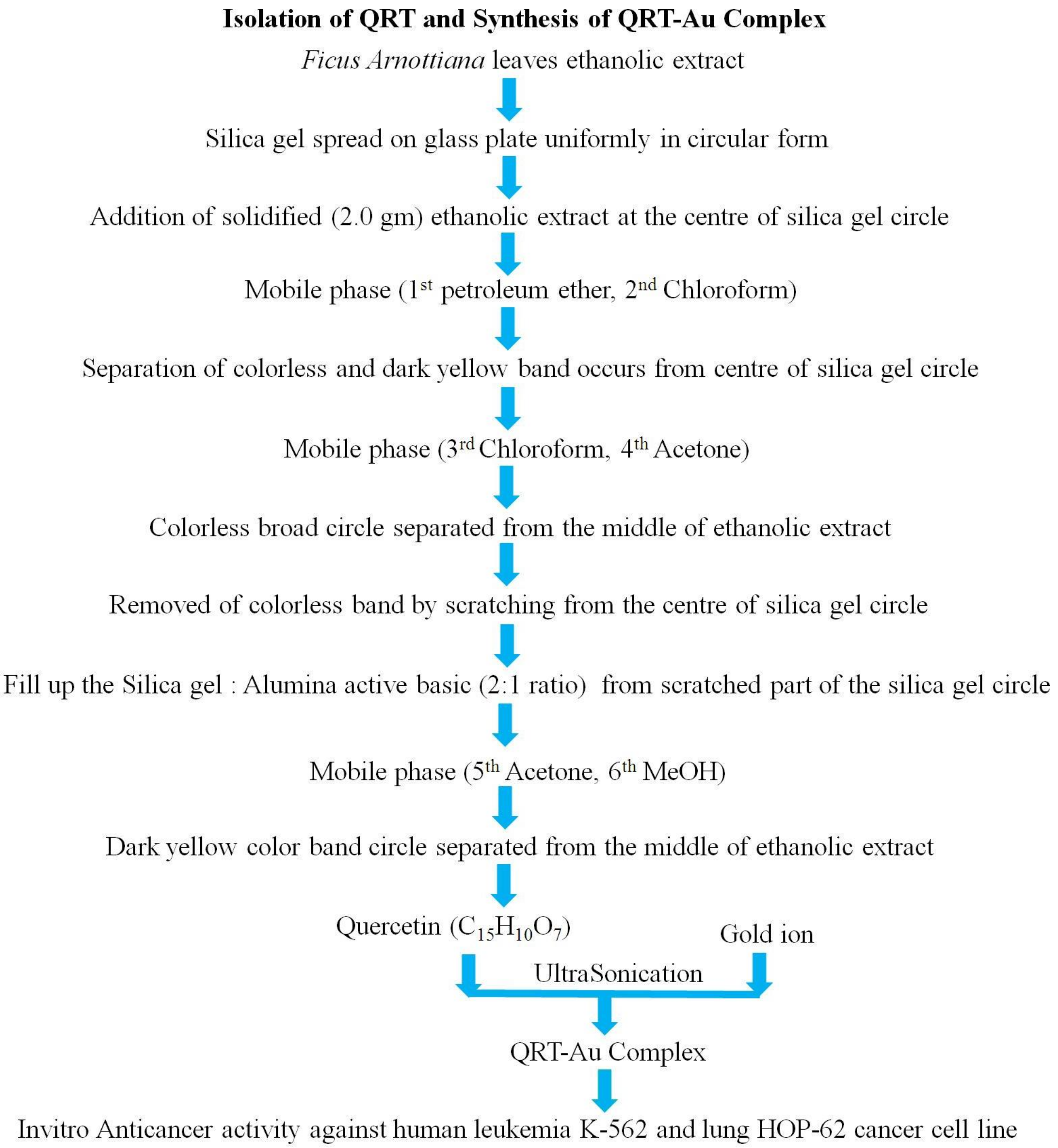

Fig. S1 Flow sheet diagram of isolation of QRT and synthesis of QRT-AuQDS.

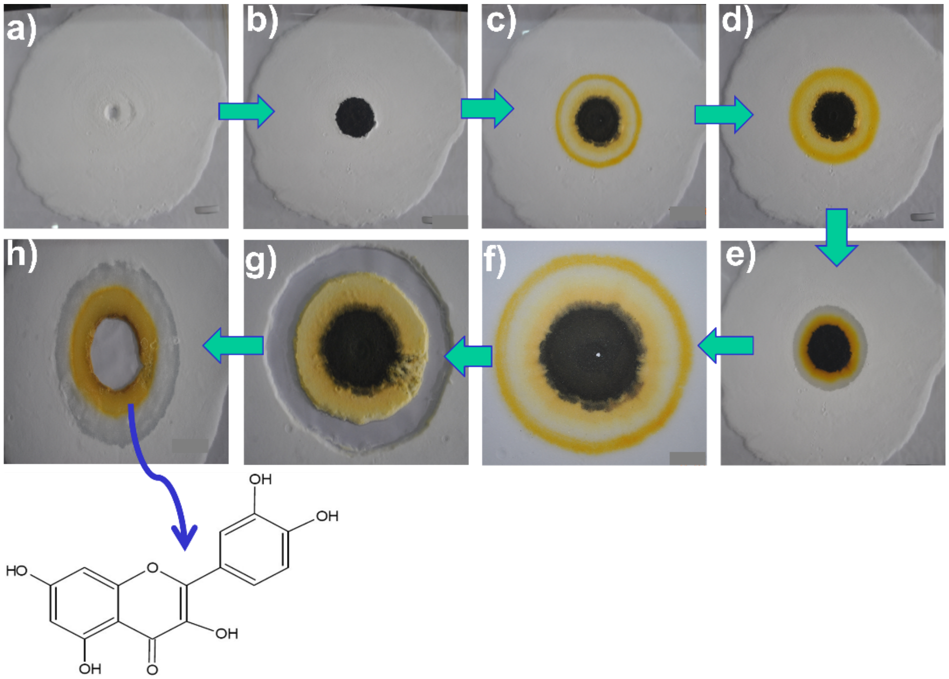

Fig. S2 a) Circularly spreaded silica gel on a glass plate. **b)** Ethanol extract of *F. Arnottiana* leaves were subjected at the centre of the CSSGLT. **c)** 1st mobile phase (petroleum ether), separation of dark yellow colored band occurred from centre of silica gel circle. **d)** 2nd mobile phase (petroleum ether and chloroform in 1:1 ratio), was used and dark yellow colored band was separated from centre of silica gel circle. **e)** 3rd mobile phase (Chloroform) was used to separate yellow colored broad circle from the middle of ethanol extract. f) 4rth mobile phase (chloroform and acetone in 2:1 ratio) was used to separate colorless broad circle from the middle of ethanol extract. **g)** Colorless part was scratched and picked up from the spreaded silica gel and the scratched area was filled up by aluminum oxide active base. **h)** 5th mobile phase (acetone and methanol in 2:1 ratio) was used and dark yellow colored circular band was separated from middle of ethanol extract.

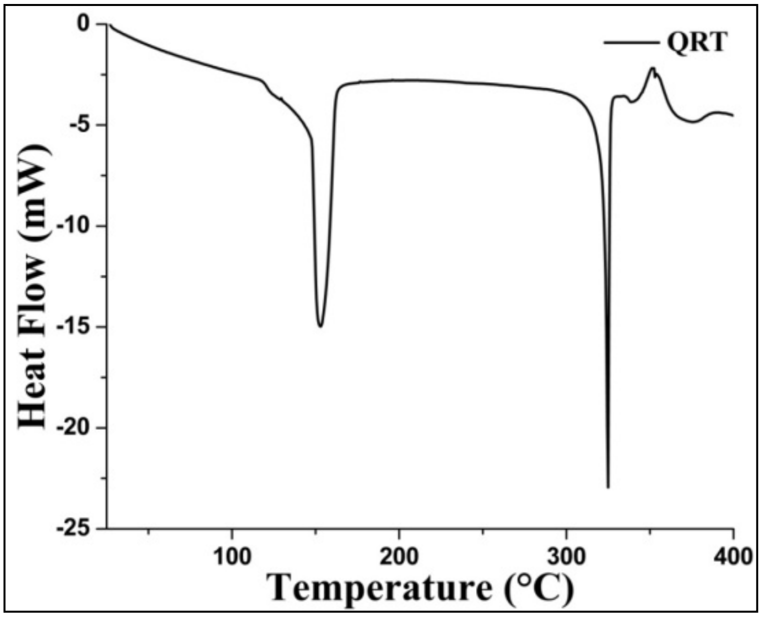

Fig. S3 DSC thermogram of QRT

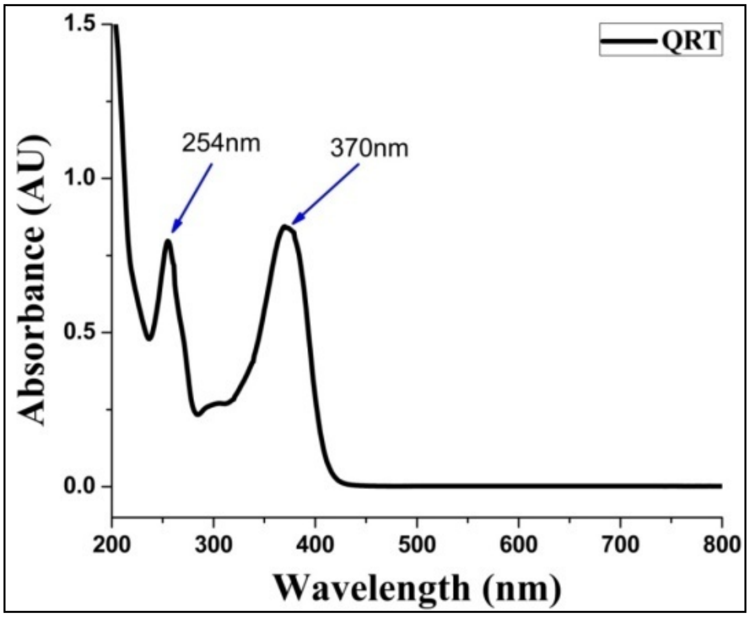

Fig. S4 UV-Vis absorption spectrum of QRT.

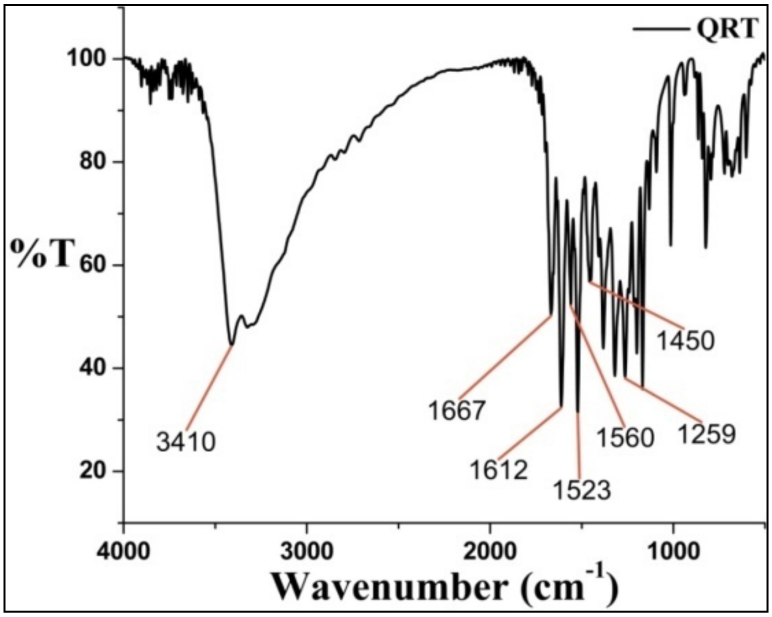

Fig. S5 FTIR spectrum of QRT.

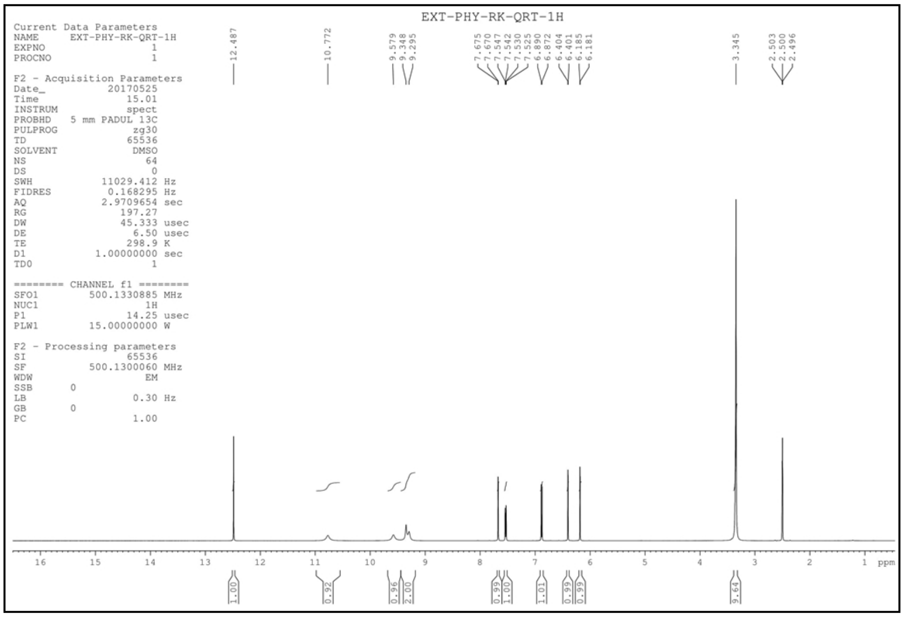

Fig. S6 ^{1}H NMR spectrum of QRT

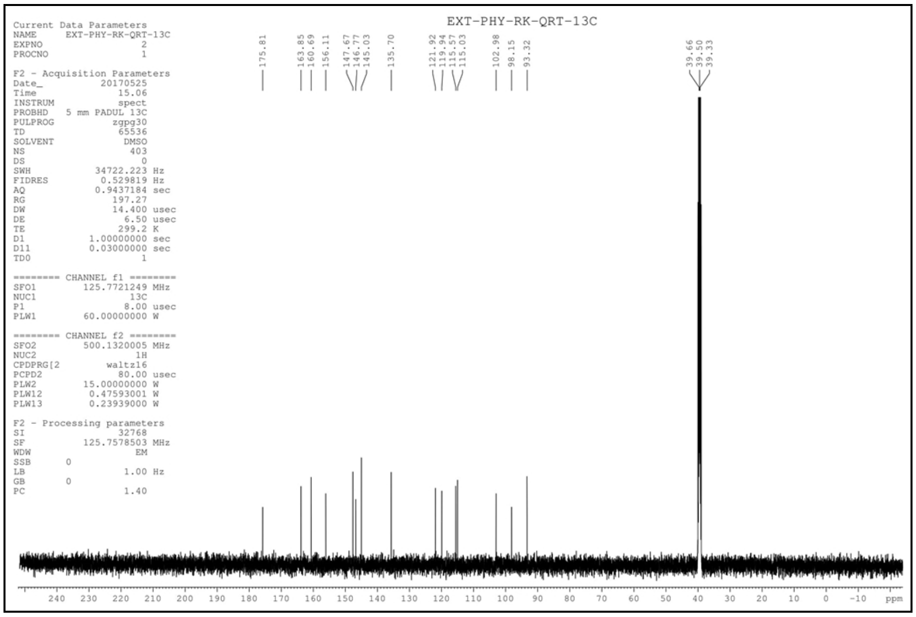

Fig. S7 ^{13}C NMR spectrum of QRT

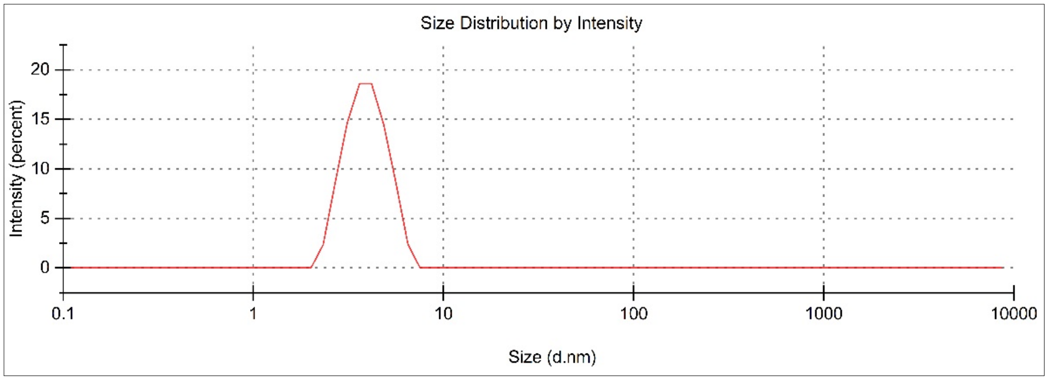

Fig.S8 DLS histogram of colloidal QRT-AuQDs (c) Zeta potential.

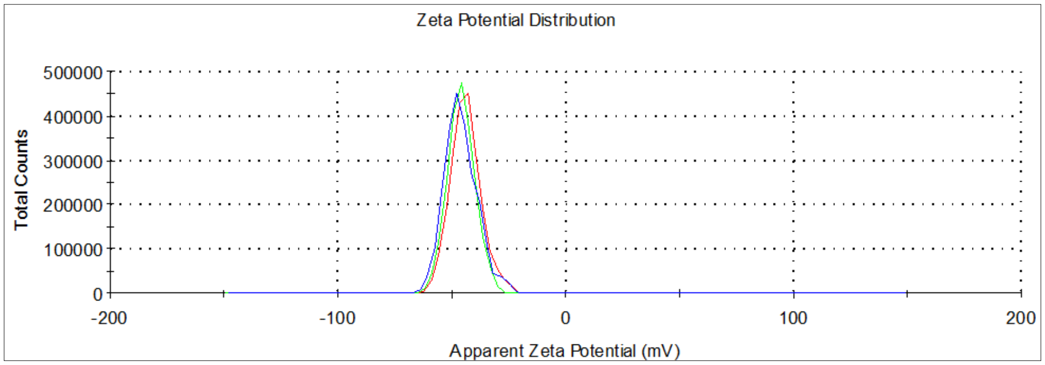

Fig.S9 Zeta potential of QRT-AuQDs

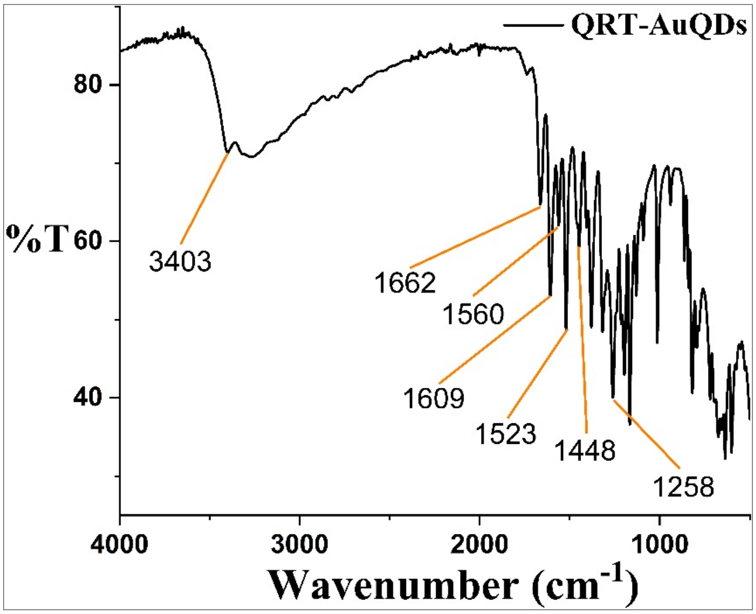

Fig. S10 FTIR spectrum of QRT-AuQDs.

Table S1 Human Lung HOP-62 cancer cell line activity of QRT and QRT-AuQDs.

	Human Lung Cancer Cell Line HOP-62							
	% Control Growth							
	Molar Drug Concentrations							
	Experiment 1				Experiment 2			
Conc.	10^{-7}	10^{-6}	10^{-5}	10^{-4}	10^{-7}	10^{-6}	10^{-5}	10^{-4}
QRT	72.6	62.5	-16.4	-18.8	26.5	51.4	-17.7	-20.2
QRT-AuQDs	69.2	58.2	-26.0	-26.8	18.2	40.1	-20.9	-22.6
ADR	84.7	40.0	-10.5	-25.9	-2.2	14.0	-26.6	-38.7
	Experiment 3				Average Values			
Conc.	10^{-7}	10^{-6}	10^{-5}	10^{-4}	10^{-7}	10^{-6}	10^{-5}	10^{-4}
QRT	-30.3	16.0	-15.5	-25.7	22.9	43.3	-16.5	-21.6
QRT-AuQDs	-4.5	48.4	-28.7	-28.8	27.8	48.9	-25.2	-26.1

ADR	-26.8	-8.8	-25.3	-39.1	18.6	15.1	-20.8	-34.5

Table S2 Human Leukemia K-562 cancer cell line activity of QRT and QRT-AuQDs.

	Human Leukemia Cancer Cell Line K-562							
	% Control Growth							
	Molar Drug Concentrations							
	Experiment 1				Experiment 2			
Conc.	10^{-7}	10^{-6}	10^{-5}	10^{-4}	10^{-7}	10^{-6}	10^{-5}	10^{-4}
QRT	66.6	57.9	53.2	29.1	54.5	42.4	41.0	23.6
QRT-AuQDs	45.1	49.1	27.1	18.9	37.2	34.6	-20.4	10.4
ADR	26.5	17.6	13.1	18.0	27.9	27.3	19.2	34.9
	Experiment 3				Average Values			
Conc.	10^{-7}	10^{-6}	10^{-5}	10^{-4}	10^{-7}	10^{-6}	10^{-5}	10^{-4}
QRT	40.5	55.7	46.0	23.8	53.9	52.0	46.7	25.5
QRT-AuQDs	33.3	48.2	30.5	24.1	38.5	44.0	26.0	17.8
ADR	34.5	47.1	34.6	16.8	29.6	30.6	22.3	23.3
